# Heterogeneous Multiphase Microstructure Formation through Partial Recrystallization of a Warm-Deformed Medium Mn Steel during High-Temperature Partitioning

**DOI:** 10.3390/ma15207322

**Published:** 2022-10-19

**Authors:** Saeed Sadeghpour, Vahid Javaheri, Mahesh Somani, Jukka Kömi, Pentti Karjalainen

**Affiliations:** Centre for Advanced Steels Research, Materials and Mechanical Engineering, University of Oulu, 90014 Oulu, Finland

**Keywords:** medium Mn steel, partial recrystallization, retained austenite, partitioning, warm deformation

## Abstract

A novel processing route is proposed to create a heterogeneous, multiphase structure in a medium Mn steel by incorporating partial quenching above the ambient, warm deformation, and partial recrystallization at high partitioning temperatures. The processing schedule was implemented in a Gleeble thermomechanical simulator and microstructures were examined by electron microscopy and X-ray diffraction. The hardness of the structures was measured as the preliminary mechanical property. Quenching of the reaustenitized sample to 120 °C provided a microstructure consisting of 73% martensite and balance (27%) untransformed austenite. Subsequent warm deformation at 500 °C enabled partially recrystallized ferrite and retained austenite during subsequent partitioning at 650 °C. The final microstructure consisted of a heterogeneous mixture of several phases and morphologies including lath-tempered martensite, partially recrystallized ferrite, lath and equiaxed austenite, and carbides. The volume fraction of retained austenite was 29% with a grain size of 200–300 nm and an estimated average stacking fault energy of 45 mJ/m^2^. The study indicates that desired novel microstructures can be imparted in these steels through suitable process design, whereby various hardening mechanisms, such as transformation-induced plasticity, bimodal grain size, phase boundary, strain partitioning, and precipitation hardening can be activated, resulting presumably in enhanced mechanical properties.

## 1. Introduction

Medium Mn steels (MMnS) containing 3–12 wt.% Mn have attracted increasing interest as potential candidates for automotive applications because of an excellent strength–ductility–toughness balance along with inexpensive alloying [1,2]. The key factor in governing a good strength–ductility combination lies in controlling properly the volume fraction and stability of retained austenite (RA). Considerable efforts have since been devoted to finetuning the balance of mechanical properties through suitable composition and process designs [3,4,5,6], thus leading to appropriate control of grain size [5,6] as well as phase fractions [7], and their morphology and heterogeneity [4].

MMnS are usually processed using intercritical annealing treatment (IAT), mostly starting from a martensitic microstructure as the dominant constituent [8]. The IAT determines the chemical composition, volume fraction, and grain size of RA [9,10,11]. However, in steels with a low Mn content, a long IAT time would be required to enrich austenite with an appropriate fraction of Mn to achieve a preferred fraction of stabilized RA in the final microstructure, due to the sluggish kinetics of Mn partitioning [10]. Such a prolonged IAT inevitably leads to coarsening of the recrystallized ultrafine-grained ferrite/martensite matrix and substantially deteriorates the strength of MMnS [12,13]. Therefore, a relatively high annealing temperature, usually above 600 °C (slightly below or above A_r1_ temperature), is needed to accelerate Mn partitioning, while avoiding or at least delaying the loss of carbon through cementite formation. As a result, the hardness of the martensite phase decreases significantly due to extensive tempering following the reduction of both the dislocation density as well as solid solution carbon content during IAT. This makes the further strengthening of intercritically annealed MMnS difficult at a constant volume fraction of RA. Therefore, it is necessary to accelerate austenite reversion and/or partitioning using different methods.

The process of quenching and partitioning (Q&P) is normally applied in the temperature range of 250–450 °C to stabilize austenite down to room temperature (RT) through the partitioning of carbon from supersaturated martensite to untransformed austenite [14,15,16]. Q&P processing has successfully been applied to MMnS resulting in a microstructure comprising low-carbon martensite and carbon-enriched RA [17]. The martensite contributes to high strength, while the RA improves ductility. Recently, it has been shown [18,19] that the Q&P concept could be extended to higher temperatures to enable the partitioning of substitutional alloying elements, such as Mn in addition to C partitioning.

One effective method to accelerate the partitioning of alloying elements could be the introduction of a high density of crystal defects in the microstructure. The most common processing that is applied to MMnS includes hot-rolling followed by cold-rolling and IAT [8,20]. On the other hand, the microstructure of hot-rolled MMnS after IAT comprises lath-shaped martensite and austenite. For cold-rolled MMnS, recrystallization of deformed martensite along with austenite formation during IAT leads to the formation of an equiaxed ultrafine-grained ferritic/austenitic microstructure [21]. Kim et al. [22] reported that after cold rolling, the partially recrystallized ferrite grains and a combination of several strengthening mechanisms could result in high yield strength and enhanced work hardening of the MMnS. Bai et al. [23] suggested that the utilization of non-recrystallized austenite and recrystallized ferrite in a cold-rolled MMnS is an effective method to produce steel of ultrahigh strength (1000–1500 MPa) and excellent ductility (>40%). They showed that non-recrystallized austenite with low mechanical stability can strengthen the MMnSs through the transformation-induced plasticity (TRIP) effect, while the recrystallized ferrite can enhance ductility through sustainable plastic deformation [23]. In addition, the coupled influence of grain refinement, dislocation strengthening, and precipitation contributes to an increase in yield strength. In general, the initial microstructure strongly affects the final phase morphology, so that both the lath and globular morphologies of RA can be observed [8,20,24].

In addition to the hot and cold deformation routes, the warm rolling process can also emerge as an alternative method to achieve the required properties. There are, however, very few research works on the warm-deformed MMnS [25,26,27,28,29]. Chang et al. [28] and Li et al. [29] demonstrated that the warm stamped 0.1C-5Mn steel exhibited more prominent work hardening, larger ductility, and better impact toughness compared to the boron-alloyed 22MnB5 Fe-Mn steel. He et al. [26] have recently shown that warm deformation of an MMnS at 300 °C following IAT enhanced the stability of RA by creating intense dislocation networks.

The presence of prior austenite in the initial microstructure of MMnS and its fraction can also affect the partitioning process and austenite transformation from martensite during IAT and accordingly the final microstructure and mechanical properties. Liu et al. [27] reported that in a martensite–austenite dual-phase structure of 0.47C-10Mn-2Al-0.7V steel (concentrations are in wt.%), warm rolling (50% reduction at 750 °C) promoted ferrite transformation during IAT at 625 °C, in addition to austenite reversion, so that these two transformations provided a heterogeneous and multiphase microstructure susceptible to enhanced strain hardening. Tsuchiyama et al. [30] demonstrated that the presence of pre-existed austenite in 0.1C-5Mn-1.2Si steel led to the formation of fresh martensite (FM) during final cooling, which improved the tensile strength of the steel. Ding et al. [31] studied the effect of pre-existed austenite on subsequent Mn partitioning and austenite formation in a 0.2C-8Mn-2Al MMnS. They reported that 10% of pre-existed austenite accelerated the kinetics of austenite reversion and the final RA fraction could be even higher than its equilibrium value.

From the microstructure point of view, it has been shown that a heterogeneous microstructure can be very efficient in providing an enhanced strength–ductility combination in materials [27,32,33,34]. The hetero-structured multiphase microstructure facilitates stress and strain partitioning between the phases [35], in addition to the strain hardening induced by grain size gradient [36,37], and enables mechanisms such as deformation-induced martensite formation over a certain extended strain range depending on the stability of RA with heterogeneous sizes and morphologies [38]. Given that recrystallization is known to drastically decrease the strength of deformed materials [39], partial recrystallization has been widely used as a tool to preserve the strength to a certain extent, while improving ductility by imparting heterogeneous structures with soft and hard domains in various alloys [40]. Kim et al. [22] showed that the partially recrystallized ferrite grains contribute to the high yield strength of MMnSs due to the low mobility of screw dislocations. Accordingly, partial recrystallization provides an opportunity to achieve a heterogeneous microstructure in MMnS as well with the matrix consisting of both the recrystallized ferrite and non-recrystallized martensite that contributes to the enhancement of yield strength via heterogeneous deformation-induced hardening [22,41,42,43].

As concluded from the above, the design of heterogeneous microstructure in MMnS including heterogeneity in phases, morphologies, and sizes could result in a superior combination of high strength and large ductility. Though several processing strategies have since been individually investigated for accelerating the partitioning of alloying elements and promoting the heterogeneity in the final microstructure of MMnSs, incorporation of a controlled combination of these strategies, however, might seemingly be more effective in enhancing mechanical properties from the microstructure engineering perspective. In the previous work [44], a combination of factors accelerating the stabilization of austenite, including pre-existed austenite, pre-deformation at 250 °C, and high-temperature partitioning was employed to create a refined microstructure in a 0.31C-4Mn-2Ni-0.5Al-0.2Mo (in wt.%) steel. In the present work, a modified process is proposed to produce a heterogeneous multiphase microstructure consisting of martensite, recrystallized ferrite, pre-existed austenite, reverted austenite, and carbides by warm deformation at a higher temperature promoting partial recrystallization of ferrite and austenite reversion in an MMnS. The strategy is composed of three steps: (1) quenching of the austenitized specimen to a temperature below martensite start temperature (M_s_) to produce a martensitic/austenitic structure, (2) warm deformation of the dual-phase alloy at a temperature above M_s_ to create high densities of crystal defects and potential nucleation sites for new phases in the microstructure, (3) annealing of the deformed microstructure to recover and partially recrystallize the martensite grains and to achieve the desired fraction of stabilized austenite, and finally cooling to RT.

## 2. Experimental Procedure

**Figure 1:**
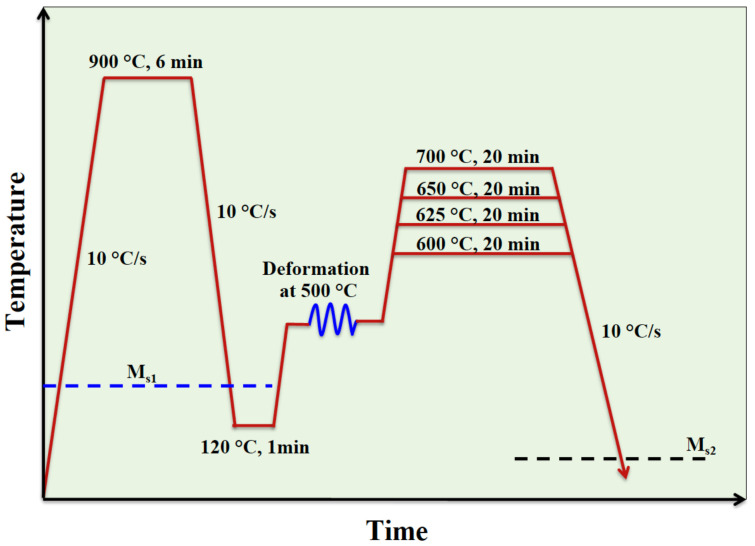
Sketch of the applied processing route.

An MMnS with a chemical composition of Fe-0.31C-4Mn-2Ni-0.42Al-0.21Mo (in wt.%) was used in the present study. A 200 mm × 80 mm × 55 mm piece of the vacuum induction melted ingot was cut and homogenized at 1200 °C for 2 h and then hot-rolled to 11 mm in thickness. Cylindrical specimens with a diameter of 6 mm and a height of 9 mm were cut with the axis transverse to the rolling direction. The samples were used for processing and dilatometry tests on a Gleeble 3800 thermomechanical simulator. The specimens were first reheated at 900 °C, held for 6 min, then cooled to 120 °C to achieve a microstructure of ~75% martensite and ~25% untransformed austenite. Then, the martensitic–austenitic specimens were reheated to 500 °C where a compressive true strain of ~0.4 was applied at a constant true strain rate of 0.1. The deformed samples were immediately heated to the annealing temperatures of 600, 625, 650, and 700 °C and soaked for 20 min and subsequently cooled down to RT (hereafter referred to as samples Def-A600, Def-A625, Def-A650, and Def-A700, respectively). A constant rate of 10 °C/s was used for all the heating and cooling steps during the processing cycle. Figure 1 illustrates a schematic of the applied process.

To characterize the microstructures, the processed specimens were sectioned along their compression axis and prepared using standard metallographic methods. A field emission scanning electron microscope (FE-SEM) with electron backscatter diffraction (EBSD) and a scanning/transmission electron microscope (STEM/TEM) were used for microstructure observations. The specimens were finally polished with a colloidal solution of silica suspension in H_2_O_2_ for EBSD analysis. EBSD scans were conducted with a step size ranging from 50 to 20 nm. For STEM/TEM observations, 3 mm discs were first ground to a thickness of 100 μm and then electrochemically polished with a solution of 5% perchloric acid and 95% ethanol using a twin-jet polisher operated at −15 °C. Interesting microstructural features were analyzed in respect of chemical composition via energy-dispersive X-ray spectroscopy (EDS) mapping in the STEM. Phase fraction was determined using X-ray diffraction (XRD) with Co-Kα radiation (λ = 0.179 nm). Vickers hardness measurements were carried out on all specimens using a 5 kg load.

## 3. Results and Discussion

**Figure 2:**
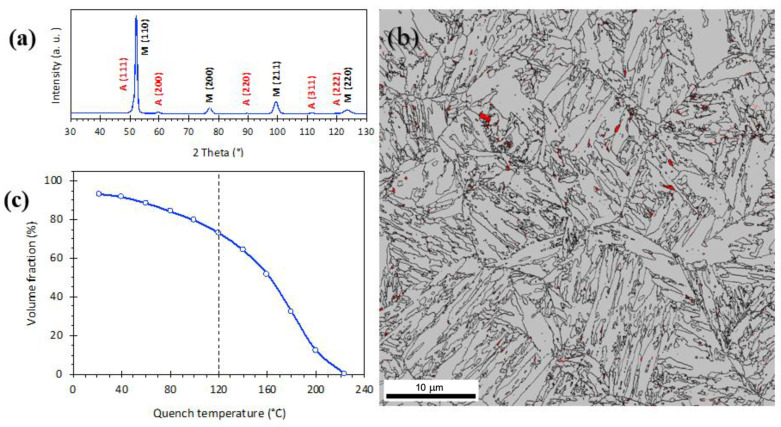
(**a**) XRD pattern of the sample quenched to RT after annealing at 900 °C, indicating martensite (M) and weak austenite (A) peaks, (**b**) corresponding EBSD phase map (austenite in red and martensite in grey), and (**c**) volume fraction of martensite at different quench temperatures.

According to the dilatometry results, austenite transformation start (A_s_) and finish (A_f_) temperatures, and the martensite start temperature (M_s_) of the studied steel were measured to be ~713 °C, ~802 °C, and 223 °C, respectively. According to XRD results shown in Figure 2a, the microstructure following reaustenitization at 900 °C and quenching to RT was mainly (93%) martensitic. The EBSD phase map of the corresponding sample confirmed that the microstructure essentially consisted of martensite and a very small amount of retained austenite, as shown in Figure 2b. The average prior austenite grain size was measured to be around 10 µm. Figure 2c shows the variation of martensite fraction as a function of quench temperature as calculated from the dilatation curve (not shown here) using the lever rule. The dashed line in Figure 2c indicates that the volume fraction of martensite is around 73% after quenching to 120 °C, so the volume fraction of pre-existed austenite is estimated to be about 27%.

**Figure 3:**
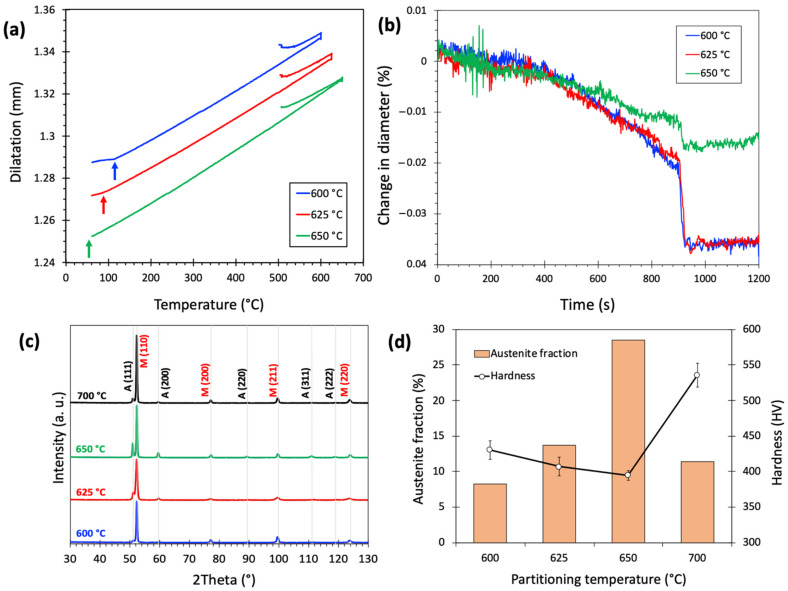
(**a**) Dilatation curves of the Def-A600, Def-A625, and Def-A650 samples, (**b**) corresponding curves of the change in diameter vs holding time, (**c**) corresponding XRD patterns showing the presence of austenite (A) and martensite (M) phases in the final microstructure and (**d**) corresponding austenite fractions and hardness values.

Figure 3a presents the dilatation curves of the Def-A600, Def-A625, and Def-A650 samples. A secondary martensite formation was detected around 120 °C (M_s2_ temperature) during final cooling after annealing at 600 °C, as shown by the arrow. It is expected that after initial quenching, the M_s_ temperature of the untransformed austenite to be just the same as the quench-stop temperature (i.e., 120 °C). Therefore, it can be concluded that in the process of warm deformation, subsequent heating, and isothermal holding at 600 °C, the austenite was not stabilized at all. In spite of this, with increasing the annealing temperature to 625 °C and 650 °C, the M_s2_ temperatures of the untransformed austenite decreased to 90 °C and 60 °C, respectively, indicating the stabilization of austenite to some extent. Figure 3b shows the change in the diameter of the samples as a function of holding time at different annealing temperatures. The curves indicated an initial contraction up to 850 s, followed by a plateau until the end of isothermal holding. The magnitude of total contraction decreased with increasing annealing temperature from 600 °C to 650 °C. However, after a certain holding time, some microstructural changes occurred causing expansion in competition with the contraction caused by partitioning and/or austenite formation. Several processes, such as the recovery and recrystallization of deformed martensite, austenite formation, and precipitation of carbides can cause volume contraction during holding at a particular temperature. In contrast, the transformation of austenite to phases such as ferrite, pearlite, and/or bainite causes volume expansion. These phase transformations that may occur during isothermal holding can affect the final volume fraction and the chemical stability of RA. The expansions observed here are most probably associated with the austenite decomposition, which has also been investigated in the previous work [44]. The decomposition processes may reduce the austenite fraction, so that if less new austenite forms, the amount of RA will decrease despite its higher stability.

Figure 3c,d show the XRD patterns of the samples annealed at different temperatures and the corresponding volume fractions of RA calculated based on the integrated intensities of austenite and martensite peaks. According to calculations, the RA fraction increased from 8% to 29% with an increase in the annealing temperature from 600°C to 650 °C. This increase in RA fraction, and less new fresh martensite (FM) as illustrated in the following, and a related decrease in dislocation density of tempered martensite (TM) due to higher partitioning temperature led to a decrease in hardness from 430 HV to 396 HV. Further increase in partitioning temperature to 700 °C resulted in a decreased RA fraction to 11% and an increased fraction of FM, while the hardness value increased to 536 HV. Thus, the maximum RA fraction was detected in the sample annealed at 650 °C. The change in the RA fraction and the hardness of the sample are associated with the formation of new martensite from less-stable austenite, and this is discussed based on the microstructure.

**Figure 4:**
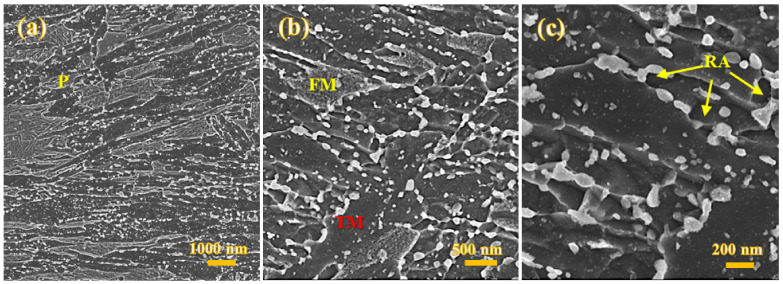
FE-SEM micrographs of the Def-A600 sample showing pearlite (P), tempered martensite (TM) some fresh martensite (FM), and several carbides on the lath boundaries and inside the laths (**a**,**b**), recrystallized ferrite and equiaxed retained austenite (RA) nucleated on carbide/ferrite boundaries and presence of very fine carbides inside the TM, ferrite, and RA grains (**c**).

As SEM micrographs in Figure 4a–c present, the microstructure of the Def-A600 sample exhibits several features inherited from primary TM, FM, pearlite (P), RA, and carbides. The lath-shaped TM (dark areas) can be characterized thanks to the presence of various carbide precipitates. Two different morphologies of TM are detected: the equiaxed fine grains, i.e., the recrystallized ferrite with size in the range of 200–300 nm (Figure 4c), and the lath morphology. Such heterogeneity in morphology can be attributed to the partial recrystallization of martensite during annealing at 600 °C. Carbide-free FM islands are somewhat lightly etched compared to TM (Figure 4c) but can still display some substructures. Some pearlite phase constituents were also detected in the microstructure as shown by arrows in Figure 4a. In addition, two lath-type and equiaxed morphologies of RA were also observed. However, TM usually contains both large and fine carbide precipitates, unlike in the case of RA, where carbides are generally fine. Though it is difficult to distinguish RA from TM using SEM images due to their similar contrast and morphology, RA can be easily identified by the EBSD phase maps. Due to more nucleation sites created during deformation, many carbides were observed along the boundaries of partially recrystallized grains with a globular morphology, as shown in Figure 4c. As mentioned, several carbides were detected with varying sizes. Large carbides with a size in the range of 80–150 nm were observed mainly on lath boundaries, while both medium-size carbides in the range of 20–50 nm and fine ~10 nm carbides were found inside the TM.

**Figure 5:**
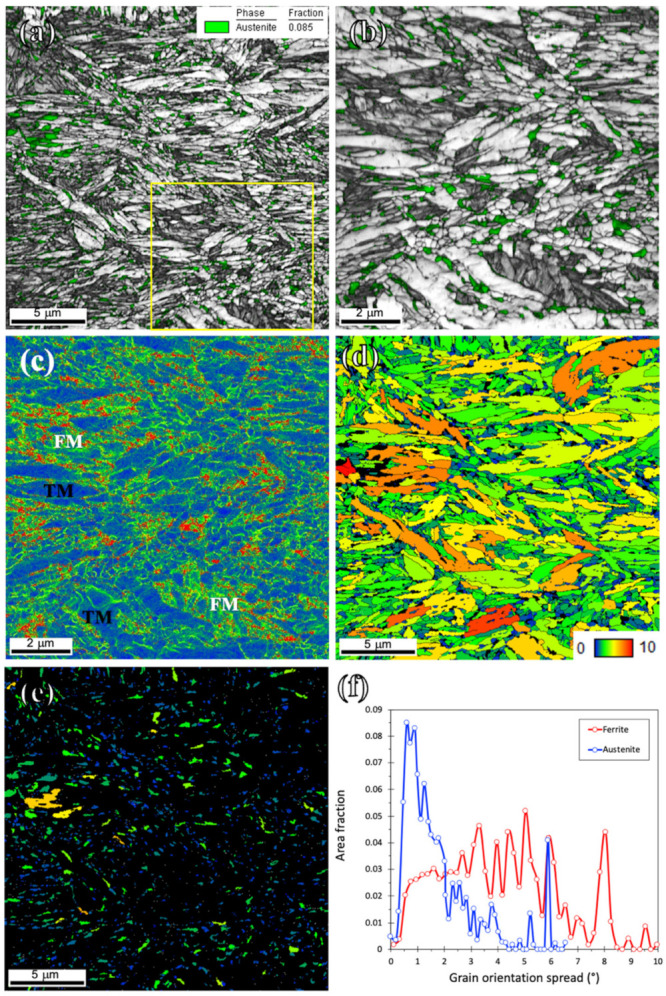
EBSD maps of the Def-A600 sample. (**a**) IQ map overlayed by phase map, (**b**) higher magnification of the marked area in (**a**), (**c**) KAM, (**d**) and (**e**) grain orientation spread (GOS) maps; and (**f**) GOS distribution for ferrite and austenite.

Figure 5a,b presents the image quality (IQ) map overlayed by a phase map plotted from the EBSD examinations carried out on the Def-A600 specimen. The RA fraction was estimated to be ~8.5% in agreement with the XRD results presented in Figure 3d. In fact, a significant amount of austenite is consumed by transforming to P and FM during holding at 600 °C and subsequent quenching. The average grain size of RA was estimated to be around 150 nm, which is markedly smaller than that of the as-received specimen (10 µm). A considerable amount of FM was observed in the microstructure, which is in agreement with the dilatometric result (Figure 3a), giving an M_s2_ temperature of 120 °C. The IQ maps in Figure 5a,b clearly show that the TM has a higher IQ value, i.e., is brighter compared to the FM due to its lower defect density. The kernel average misorientation (KAM) map in Figure 5c shows that the TM contains a low density of geometrically necessary dislocations because of dislocation annihilation during the tempering, resulting in low KAM values. In contrast, significantly higher KAM values, i.e., dislocation densities are detected in FM areas. In addition to the differences in dislocation density, the TM contains a very low amount of C due to its partitioning into the austenite, while the FM forms from C-enriched austenite rendering it to be an untempered, high-C hard phase. Therefore, the hardness of the FM formed during final quenching is expected to be higher than that of the TM formed during initial quenching to 120 °C following austenitization (Figure 3d).

Unlike in the case of cold-rolled MMnS which usually provides a fully recrystallized microstructure after IAT [21], warm rolling only promoted partial recrystallization in the ferritic matrix as a result of a comparatively lower driving force. Further, austenite had two different types of morphologies, i.e., lamellar and equiaxed. We may assume that due to the lower driving force for recrystallization in the warm-rolled matrix, the recrystallization of ferrite and its reversion to austenite took place simultaneously. Whilst reversion of martensite laths led to the formation of lamellar RA of size <200 nm, recrystallized ferrite grains transformed to equiaxed RA with size varying in the range 150–600 nm (Figure 5a,b). In addition to the new austenite formation through reversion, partial recrystallization of lamellar pre-existed austenite may produce equiaxed or lamellar morphology in RA. Therefore, both ferrite and austenite phases showed heterogeneity in terms of size and morphology.

Figure 5d,e shows the grain orientation spread (GOS) maps of ferrite and austenite of the Def-A600 sample. As the inset in Figure 5d shows, the GOS value varies from 0 (blue) to 10 (red). This blue-to-red range was set identically so that the different conditions could be directly compared. The GOS maps comparing the local orientation of each point with respect to the orientation of the neighboring points have widely been used in evaluating the degree of recrystallization [45,46,47]. As seen in Figure 5d, a considerable number of ferrite grains had a high GOS value illustrating that those grains were not fully recrystallized. In contrast, most austenite grains appearing as blue or green displayed low GOS values. However, we have to notice that even with a step size of 50 nm, orientation differences are difficult to detect by GOS in grains with a size below 200 nm. The lamellar old austenite might be partly recrystallized, but 600 °C is a very low temperature for recrystallization, even though some strain partitioning might have concentrated strain in austenite. In austenitic stainless steels, the lowest recrystallization temperature is about 700 °C [48], and in duplex stainless steel, recrystallization of austenite occurs above 800 °C (though 20% cold rolling reduction, only) [49]. Therefore, despite possibly higher energy stored in austenite owing to strain partitioning between martensite and austenite during the warm deformation, there is no real evidence for the recrystallization of austenite at 600 °C, and GOS limited resolution might lead to a wrong conclusion. Fine grains may be new equiaxed austenite nucleated during annealing, being naturally dislocation-free. The formation of new dislocation-free austenite is consistent with the KAM results, though the recrystallization of pre-existed austenite cannot be ruled out and would be the subject of future studies.

Figure 5f presents a comparison of the GOS distribution for ferrite and austenite. While the ferrite grains showed a wide range of GOS owing to the presence of both recrystallized grains along with non-recrystallized grains, the austenite grains displayed a relatively narrower distribution of GOS. Two well-separated peaks for the GOS values of both ferrite and austenite grains can be identified as corresponding to the recrystallized or new grains and deformed non-recrystallized ones. Grains with a GOS less than 2° were classified as fully recrystallized ferrite which is in agreement with the reported threshold value for GOS [45,46,47], or dislocation-free new austenite. Based on the criterion of GOS <2°, the dislocation-free fractions were determined to be 19% and 67% for ferrite and austenite, respectively.

**Figure 6:**
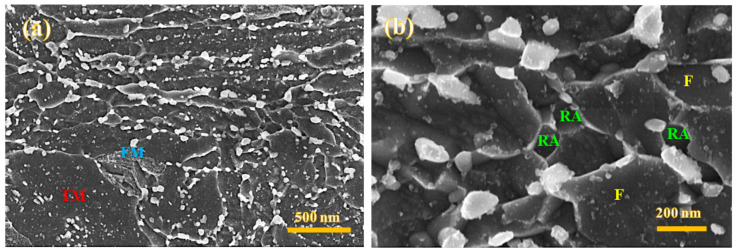
FE-SEM micrographs of the Def-A650 sample showing TM, FM, and carbides on the martensite lath boundaries and inside the laths (**a**), recrystallized ferrite (F), and equiaxed RA nucleated on carbide/ferrite boundaries along with very fine precipitates inside the F and RA grains (**b**).

The microstructure of the Def-A650 sample contained several features including TM, FM, RA, and carbides, as shown in Figure 6a. It is also obvious from Figure 6b that the recrystallized grains of the specimen partitioned at 650 °C are somewhat coarser compared to those of the Def-A600 specimen. Unlike in the case of the Def-A600 sample, the absence of pearlite in the microstructure of the Def-A650 sample suggested slow kinetics of pearlite transformation at 650 °C despite holding for 20 min. The retardation of pearlitic transformation as a result of increasing annealing temperature has also been discussed in the previous study [21]. RA grains (fraction about 28%) were mainly equiaxed, in addition to the presence of a small fraction of interlath austenite in the microstructure. Additionally, the volume fraction of carbides following partitioning at 650°C was lower than that observed in the case of the sample partitioned at 600 °C, though the average size of carbides was expectedly larger at 650 °C.

**Figure 7:**
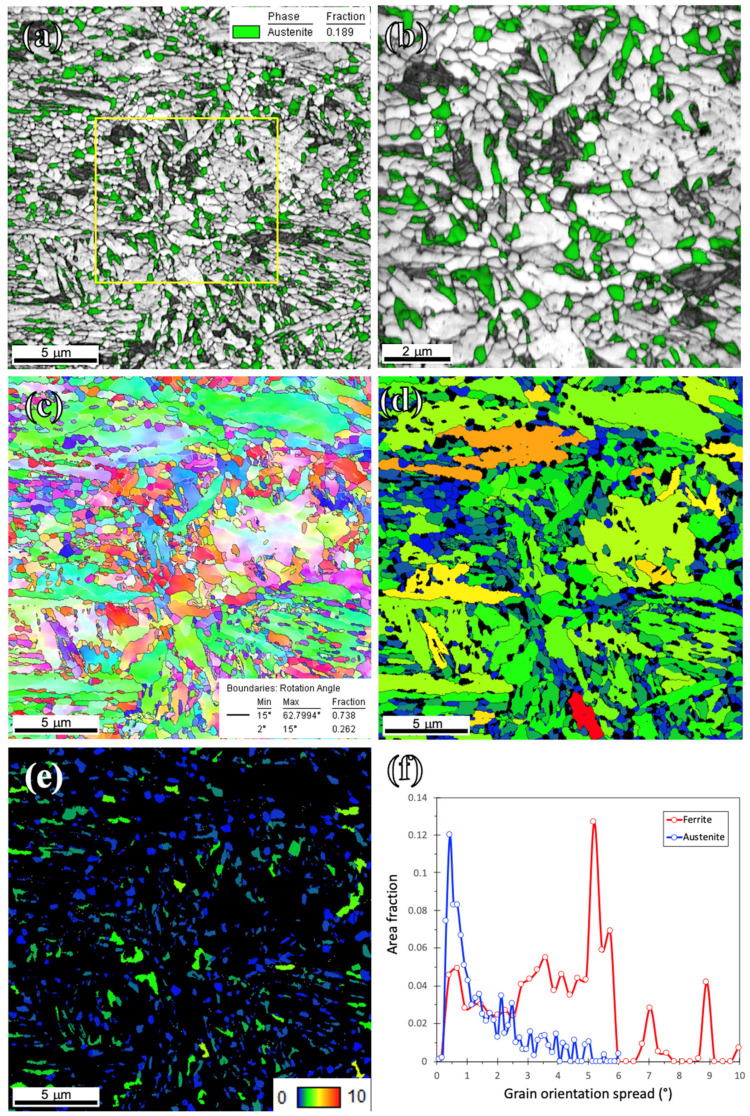
EBSD maps of the Def-A650 sample. (**a**) IQ map overlayed by phase map, (**b**) higher magnification of the marked area in (**a**), (**c**) IPF and (**d**,**e**) grain orientation spread (GOS) maps; and (**f**) GOS distribution for ferrite and austenite.

Figure 7 illustrates the EBSD maps of the Def-A650 specimen, where the RA appears mostly as equiaxed grains, along with the TM, recrystallized ferrite, and some contents of the FM. According to Figure 7a, a significantly higher fraction of RA is observed compared to that in the Def-A600 specimen, in consistence with the XRD results shown in Figure 3c. The fraction is practically the same as that of the pre-existed austenite. Though the RA grains are somewhat coarser following partitioning at 650 °C, they are more uniform in size and distribution compared to those observed in the Def-A600 specimen, as shown in Figure 5a,b. Though the TM had both equiaxed as well as lath morphologies, the area fraction of equiaxed grains increased with increasing the annealing temperature from 600 °C to 650 °C, as a result of enhanced recrystallization rate at 650 °C. The inverse pole figure map overlayed by the high-angle boundary map, shown in Figure 7c, indicates a heterogeneous grain structure containing about 74% of high-angle boundaries. Figure 7d,e shows the GOS maps of ferrite and austenite of the Def-A650 sample. According to Figure 7d, ferrite grains were not yet fully recrystallized following 20 min holding, even though the fraction of recrystallized grains was higher than that of the Def-A600 sample (Figure 5d). The recrystallized fractions of ferrite and austenite were calculated to be around 26% and 73%, respectively. The average grain size of RA increased to 600 nm with increasing the annealing temperature to 650 °C. This corroborates that a higher amount of stored energy was available in the deformed austenite compared to the deformed martensite due to strain partitioning. As shown in Figure 7f, the ferrite grains showed a wider distribution of GOS, while the austenite grains showed a narrower distribution of GOS because of a large, recrystallized fraction.

**Figure 8:**
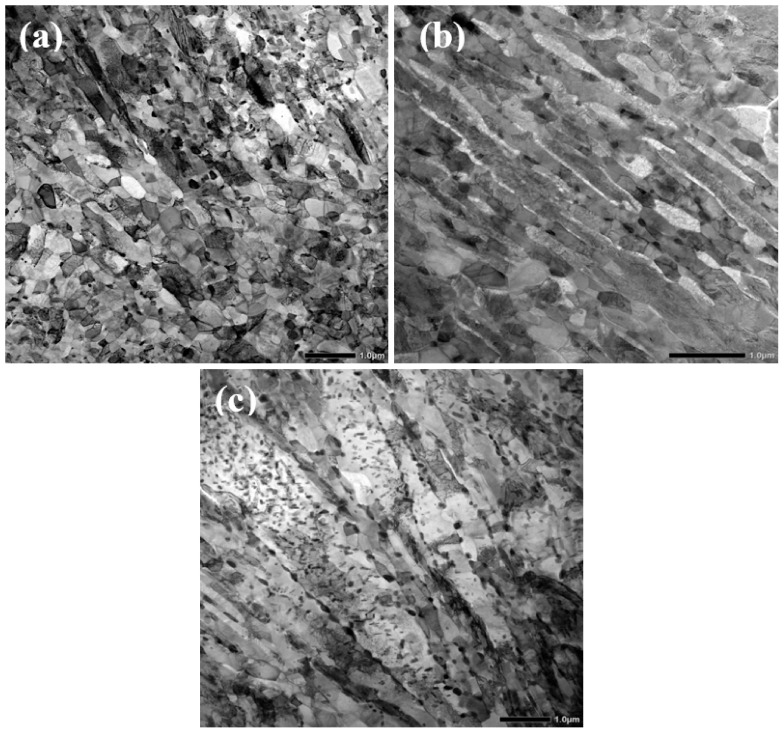
STEM images of the Def-A650 specimen. (**a**) Lath and equiaxed structure of martensite and RA phases, (**b**) two morphologies of retained austenite as films between the martensite laths, and some globular grains along with carbides on lath boundaries, (**c**) strings of large carbides on lath boundaries and spherical and rod-shaped carbides inside the TM laths.

The STEM images of the Def-A650 sample illustrate two lath-type and equiaxed morphologies for both austenite and martensite phases, as shown in Figure 8a,b. Thin interlath films of RA, surrounded by martensitic areas along with some equiaxed austenite grains can be observed as bright regions in Figure 8b. These thin RA films might be non-recrystallized pre-existed austenite or new austenite formed on non-recrystallized martensite lath boundaries. In addition to martensite and austenite, various precipitates as strings of large carbides on the lath boundaries and both spherical and rod-shaped carbides inside the TM lath were observed in the microstructure as shown in Figure 8c.

As described, the applied process resulted in a multiphase microstructure comprising TM, ferrite, RA, and carbides. Both martensite and austenite phases exhibited heterogeneous morphologies and grain structures decorated by carbides. As mentioned, these phases and morphologies could contribute to different strengthening and deformation mechanisms.

**Figure 9:**
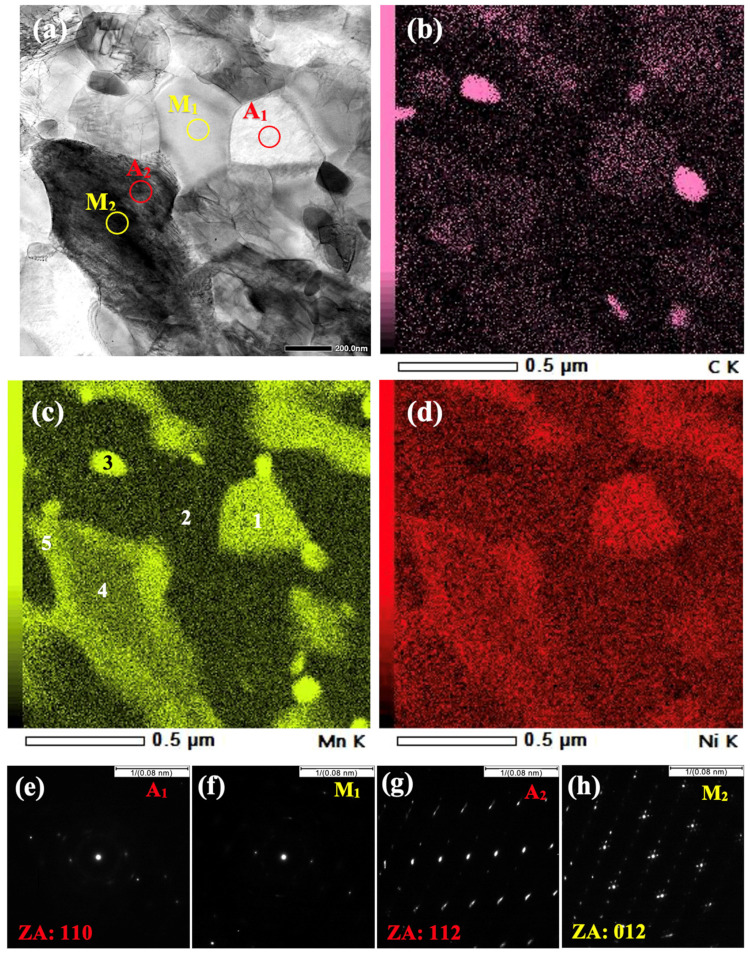
(**a**) STEM image of the Def-A650 specimen and corresponding STEM-EDS maps of (**b**) C, (**c**) Mn and (**d**) Ni distribution along with the electron diffraction patterns taken from the areas (**e**) A_1_, (**f**) M_1_, (**g**) A_2_ and (**h**) M_2_.

To investigate the partitioning of alloying elements in the final microstructure, STEM-EDS scans were conducted on the Def-A650 specimen. A typical STEM image along with corresponding distribution maps of C, Mn, and Ni for the sample are shown in Figure 9a–d, respectively. The contents of elements in the regions marked by identifying numbers in Figure 9c are listed in Table 1. The maps evidently show that C, Mn, and Ni have partitioned to specific regions and depletion in other regions. The Mn contents at points 1, 2, and 3 were measured to be 6.7%, 2.8%, and 15.7%, respectively. The equilibrium concentrations of Mn in austenite, ferrite, and cementite were calculated to be about 7.34%, 1.46%, and 13.85% at 650 °C, as reported elsewhere [44]. This suggests that point 1 in Figure 9c corresponds to the austenitic area, while point 2 is in the ferritic region, and point 3 lies in cementite. The corresponding SAED patterns in Figure 9e,f confirm that areas marked A_1_ and M_1_ in Figure 9a are distinguished as FCC and BCC structures. Since C, Mn, and to some extent Ni, partition from BCC martensite to FCC austenite, the amounts of these alloying elements in RA, especially Mn concentration, ought to be relatively higher than that in the martensitic grains. The compositions listed in Table 1 denote that there is an insignificant change in the Al and Si contents in comparison to their average value before the processing, while the C, Mn, and Ni contents are varying appreciably in different regions. This suggests that considerable partitioning of C, Mn, and Ni did take place during the process, whereas the partitioning of Al and Si to BCC ferrite was insignificant because of their lower diffusivity in FCC austenite. The partitioning of C and Mn has been reported even in a short duration of 180 s during IAT of cold-rolled MMnS in the range of 640–680 °C [50]. However, Lis et al. [51] reported no considerable partitioning of Ni, Cr, and Si in their investigation of the partitioning of various alloying elements.

In addition to typical Mn and Ni partitioning, some martensitic regions depleted of Mn and Ni that were surrounded by thin austenitic films enriched in Mn and Ni were also observed. For example, Mn concentration at point 4 was measured to be 4.7% while Mn concentration in the outer layer, (i.e., at point 5) was measured to be 7.1%. The SAED patterns presented in Figure 9g,h confirm that the exterior area close to the boundary (i.e., A_2_ in Figure 9a) has an FCC structure, while the core area (i.e., M_2_ in Figure 9a) has a BCC structure. It can be concluded that the shell regions of the larger austenite grains close to the FCC/BCC interfaces, where Mn concentration was adequately high to stabilize the austenite, retained untransformed at RT. In contrast, core regions that were Mn-lean transformed to fresh martensite during the cooling to RT. It is worth mentioning that the STEM samples were prepared at −15 °C indicating that RA was stable even until this temperature. The average thickness of the Mn-enriched austenite shell was measured to be around 150–200 nm. Given that the contents of C, Mn, and Ni in FM were measured to be higher than those in TM but lower than those in RA, it was possible to distinguish the TM, FM, and RA in the microstructure, as shown in Table 1 for the selected locations.

A similar formation of martensite inside austenite has also been reported in 5Mn-0.1C steel during cooling from the intercritical region [30]. It has been shown that the content of Mn inside the core region, can be close to its average level in the material [31], which is in good agreement with the present observations. The present results indicate that despite the higher Mn concentration in the core of the prior austenite (4.7%) in comparison to its average value in the bulk material (4%), the partitioning of Mn and Ni was still inadequate to fully stabilize austenite and only a thin shell of austenite with a thickness of 150–200 nm, where the Mn content is higher (7.1%), was stabilized. This is ascribed to the fact that the annealing at 650 °C for 20 min was too short for adequate partitioning of Mn to fully stabilize austenite because of the slow diffusion of Mn in the austenite phase. In contrast, a large area of ferrite was depleted of Mn (e.g., 2.5–3% Mn) due to the higher diffusion rate in ferrite.

The authors recently showed [44] that the prior deformation can effectively improve the Mn partitioning distance by creating high densities of dislocations as easy diffusion paths. However, due to 20 min partitioning at 650 °C, some of the grains coarsened beyond 500 nm suggesting that Mn partitioning may not be enough to stabilize large austenite grains, as their core regions transformed to FM during the cooling to RT. According to previous results [44], the critical Mn concentration to stabilize austenite in the studied steel was found to be around 5.5%. However, to consider the partitioning effect of other alloying elements on austenite stabilization, the M_s_ temperature was calculated for different grains. On the other hand, C diffuses several hundred times faster than substitutional elements such as Mn and Ni, and its equilibration is completed at the very beginning of the heating so that the effect of deformation on carbon diffusion rate is not detectable irrespective of the partitioning duration.

During IAT, the recovery and recrystallization of martensite occur before its reversion to austenite and the recrystallization is enhanced with high stored energy provided by deformation. The effect of deformation on the accelerated recrystallization of martensite before its reversion has already been observed in dual-phase steels [52] and MMnS [53]. It has also been reported that recrystallization of ~75% cold-deformed martensite occurs in less than 5 s at a temperature around A_c1_ [54]. In the present work, a smaller deformation strain of 40% at a higher temperature of 500 °C was applied, so the stored energy is much lower compared to that generated in cold deformation. Because of the lower driving force in warm deformation, the recrystallization process is slower. The recrystallization of martensite results in fine ferrite grains with low dislocation density. However, with reduced dislocation density, accelerated diffusion processes cannot operate to transport adequate Mn atoms to desired diffusion distances in austenite [44].

It has been shown that the austenite reversion in martensite–austenite structure can start at a temperature below A_c1_ [44,55,56]. The equilibrium thermodynamic calculations also indicate that austenite might form at temperatures as low as 492 °C [44]. This can be ascribed to the pre-existed austenite so that the increase in austenite fraction results from the growth of pre-existed austenite rather than by the nucleation of new grains. According to Ding et al. [31], in the presence of prior austenite, both the growth of pre-existed austenite and the nucleation of new austenite grains may take place during intercritical annealing.

To clarify the influence of partial recrystallization on microstructural evolutions, some salient microstructural characteristics obtained in the present study (the sample deformed at 500 °C) and in the previous work [44] (a non-deformed and a sample deformed at 250 °C) are compared as listed in Table 2. The main difference concerns the recrystallization fraction of ferrite which was much smaller in the specimen deformed at 500 °C. The lower recrystallized fraction is obviously due to the reduced stored energy following deformation at the high temperature (500 °C), also resulting in a heterogeneous structure with a broad grain size distribution. Though there is uncertainty about the recrystallization of austenite due to new austenite formation, still the sample deformed at 500 °C showed a lower fraction of dislocation-free grains. The sample deformed at 500 °C exhibited a comparatively larger average grain size and a higher number fraction of grains with core–shell structure following partial recrystallization resulting in a slightly lower fraction of stabilized austenite and the formation of more FM. The average M_s_ temperatures and stacking fault energies (SFE) of RA in various samples were estimated using the following equations [57,58]:

M_s_ = 517 − 423C − 30.4Mn − 7.5Si + 30Al (1)

SFE = 1.2 + 1.4 (Ni + 0.5Mn + 0.3Cu + 30C) + 0.6 (Cr + 2Si + 1.44B) (2)

The M_s_ and SFE values listed in Table 2 indicate that the RA in the sample deformed at 500 °C had lower stability in comparison to that in the sample deformed at 250 °C and non-deformed sample. This comparison indicates that by varying the deformation temperature, it is possible to vary the microstructure stability and heterogeneity in terms of size, distribution, phase fractions, morphology, and its stability.

**Figure 10:**
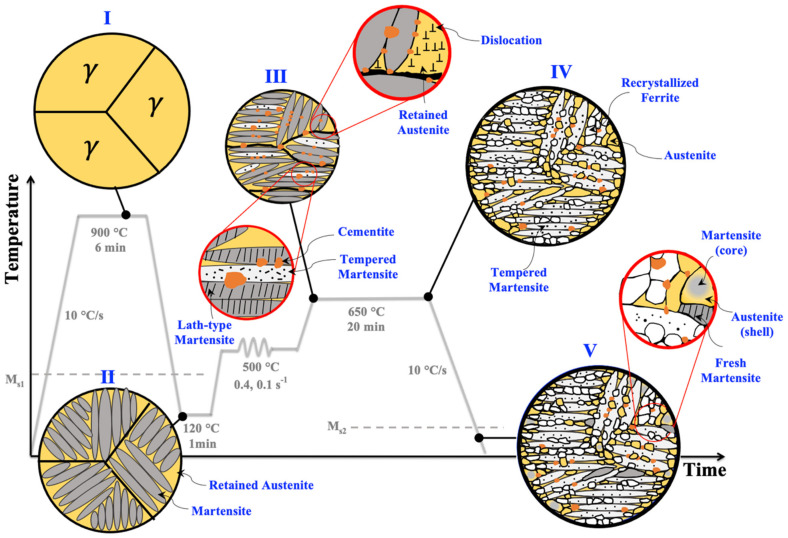
Schematic illustration of the microstructure evolution during the annealing (I), quenching to 120 °C (II), warm deformation at 500 °C (III), and IAT at 650 °C (IV) resulting in a heterogeneous multiphase microstructure (V).

Figure 10 schematically illustrates the progress of microstructural evolution during the processing of present MMnS under the application of warm deformation and subsequent partitioning annealing. The initial microstructure, which is mainly martensitic, transforms to a fully austenitic structure with a large grain size after annealing at 900 °C for 6 min (step I). Following quenching to 120 °C, a microstructure consisting of 73% martensite and 27% austenite is achieved (step II). As a result of warm deformation at 500 °C, a high dislocation density is created both in austenite as well as martensite phases (step III). Strain partitioning to the softer austenite phase makes austenite grains far more strained meaning acquisition of a higher dislocation density. During subsequent IAT at 650 °C for 20 min, both the formation of new austenite and partial recrystallisation of martensite take place along with partial carbide dissolution (step IV). During the final cooling to RT, some austenitic regions with lower chemical stability transform to FM creating a core–shell structure in addition to the bulky FM regions (step V). The applied processing route results in a heterogeneous multiphase microstructure comprising coarse-grained TM, fine recrystallized ferrite, ultrafine RA, FM, and fine carbides. The grain refinement along with C, Mn, and Ni partitioning promotes higher stability of RA grains that are envisaged to provide pronounced TRIP and/or TWIP effects and consequently enhance the strain hardening rate and ductility.

## 4. Conclusions

A new processing route was employed to achieve a highly refined, multiphase, and heterogeneous microstructure in a medium Mn steel. Based on the detailed microstructural analysis, the main conclusions are summarized as follows:

1. The processing route comprising step quenching to 120 °C, warm deformation at 500 °C, and high-temperature partitioning at 650 °C for 20 min results in the occurrence of various desired microstructural evolutions such as martensite tempering and its partial recrystallization, carbide precipitation, partitioning of elements and austenite reversion.
2. The desired multiphase and heterogeneous microstructure of the studied medium Mn steel was composed of several phases including lath-shape tempered martensite, recrystallized ferrite, pearlite, 29% of ultrafine retained austenite (in both lath-type and equiaxed morphologies), some fresh martensite and undissolved carbides.
3. A core–shell structure of Mn-lean fresh martensite surrounded by Mn-rich austenite film was detected in the final microstructure of the samples. Despite warm deformation, during the intercritical annealing at 650 °C for 20 min, Mn can diffuse only to a short distance, and in the case of austenite grain size larger than the maximum diffusion distance, an Mn-rich layer around an Mn-lean core was created. The peripheral area of Mn enrichment was stabilized, while the core area tended to transform into fresh martensite during final cooling.
4. Employing a warm deformation strain of 40% at 500 °C provided a microstructure exhibiting a wide ferrite grain size range, in addition to a moderate level of recrystallization (i.e., 26%) compared to the microstructural features noticed earlier in the case of samples either deformed at 250 °C or without any deformation. In contrast, the austenite grains displayed a high fraction (i.e., 73%) of dislocation-free grains in a narrow size range. This was attributed to the formation of new dislocation-free austenite grains, though the partial recrystallization of pre-existed austenite cannot be ruled out.
5. The calculation of M_s_ and SFE indicated that the RA in the sample deformed at 500 °C had lower stability in comparison to that in the sample deformed at 250 °C and also, the non-deformed sample. This indicates that by varying the deformation temperature, it is possible to finetune the microstructure stability and heterogeneity in terms of size, distribution, phase fraction, and morphology, known to be beneficial for mechanical properties, particularly tensile ductility.

The current results provide a better understanding of the various microstructural evolutions during the processing of MMnS and shed light on advanced microstructure design. It is shown that a judicious combination of several austenite stabilizing methods including warm deformation, pre-existed austenite, and high-temperature partitioning will be advantageous in creating a multiphase and heterogeneous microstructure, to be able to control the strengthening and deformation mechanisms, thereby enhancing the mechanical properties.

## Figures and Tables

**Table 1:** Chemical composition of the areas marked by numbers in Figure 9c measured by STEM-EDS.

Position	Mn	C	Ni	Al	Si	Mo	Phase
1	6.71	1.27	2.06	0.34	0.20	2.62	RA
2	2.84	-	0.99	0.32	0.46	0.63	F
3	15.73	9.41	0.95	0.07	0.19	0.54	Carbide
4	4.66	0.88	1.98	0.24	0.59	1.58	FM
5	7.08	1.73	2.40	0.05	0.49	-	RA

**Table 2:** Microstructural characteristics of the present steel after deformation at 500 °C (def-500) and 250 °C (def-250) and without deformation (non-def) followed by annealing at 650 °C for 20 min.

Sample	Recrystallized Ferrite Fraction (%)	Recrystallized Austenite Fraction (%)	RA Average and Range of Grain Size (μm)	Ferrite Average and Range of Grain Size (μm)	RA Fraction (%)	Ms (°C)	RA SFE (mJ/m^2^)
def-500	26	73	0.23 (0.07–0.99)	0.31 (0.11–5.51)	29	−70	45
def-250	56	78	0.24 (0.07–1.32)	0.24 (0.09–2.97)	32	−89	48
non-def	0	0	0.2–0.3 *	0.8–2 *	34	−105	48

* The lath thickness was used for this sample.
